# The Use of Alendronate Is Associated with a Decreased Incidence of Type 2 Diabetes Mellitus—A Population-Based Cohort Study in Taiwan

**DOI:** 10.1371/journal.pone.0123279

**Published:** 2015-04-13

**Authors:** Ding-Cheng Chan, Rong-Sen Yang, Chung-Han Ho, Yau-Sheng Tsai, Jhi-Joung Wang, Kang-Ting Tsai

**Affiliations:** 1 Department of Gerontology and Geriatrics, National Taiwan University Hospital, Taipei, Taiwan; 2 Department of Internal Medicine, National Taiwan University Hospital, Taipei, Taiwan; 3 Superintendent Office, National Taiwan University Hospital JinShan Branch, New Taipei City, Taiwan; 4 Department of Orthopedics, National Taiwan University Hospital, Taipei, Taiwan; 5 Institute of Medical Research, Chi Mei Medical Center, Tainan, Taiwan; 6 Institute of Clinical Medicine, College of Medicine, National Cheng Kung University, Tainan, Taiwan; 7 Department of Geriatrics, Chi Mei Medical Center, Tainan, Taiwan; 8 Department of Family Medicine, Chi Mei Medical Center, Tainan, Taiwan; Medical University Innsbruck, AUSTRIA

## Abstract

**Purpose:**

Bone remodeling has been linked to glucose metabolism in animal studies, but the results of human trials were inconclusive. Bisphosphonates may play a role in glucose metabolism through their impacts on bone remodeling enzymes. In this study, we aimed to examine the influence of alendronate usage on the incidence of type 2 diabetes mellitus (DM) among osteoporotic patients.

**Methods:**

A retrospective cohort study was designed to include osteoporotic patients without DM from a population-based cohort containing 1,000,000 subjects. Patients treated with alendronate (exposed group, N=1,011) were compared with those who received no treatment (age and gender matched non-exposed group, N=3,033). Newly diagnosed DM was identified from medical records by International Classification of Diseases, Ninth Revision, Clinical Modification (ICD-9CM) code. The incidence of DM in both groups was calculated for comparison.

**Results:**

The non-exposed group had a significantly higher incidence of DM (Odds ratio 1.21, 95% confidence interval 1.03~1.41) when compared with the exposed group. In subgroup analysis, the DM risk reduction in exposed group was only significant among those younger than 65 years and those without hypertension or dyslipidemia. Patients who were prescribed alendronate more than or equal to 3 times had demonstrated a significant reduction in DM risk.

**Conclusions:**

Our study showed alendronate might yield a protective effect for incident DM. This effect became insignificant in patients with older age, dyslipidemia or hypertension. The underlying mechanism needs further exploration with prospective data for confirmation of the observed findings.

## Introduction

Bone remodeling had been linked to glucose metabolism in animal studies[1]. Higher level of undercarboxylated Osteocalcin (ucOC), a protein produced by osteoblasts[2] and metabolized under the influence of osteoclasts during bone remodeling[3], was found to increase insulin secretion and sensitivity in mice[4].

Most human studies in the past for the association between bone and glucose metabolism were cross-sectional in design. In human trials, serum total OC was inversely associated with fasting plasma glucose, fasting insulin and insulin resistance [5, 6]. However, the association between ucOC and insulin secretion was not certain[7]. Unlike animal model, one human study found that decreased ucOC was associated with increased insulin sensitivity [8].

Bisphosphonates are anti-resorptive medications used widely to treat osteoporosis. Bisphosphonates can suppress osteoclasts’ function with significant decrease in both total OC and ucOC levels in patients under treatments [9–11]. With their effects on altering OC and ucOC levels, bisphosphonates may consequently affect glucose metabolism in human.

The association between usages of bisphosphonates and glucose metabolism is still inconclusive. A small experimental study showed that alendronate reduces the daily consumption of insulin in patients with type I diabetes mellitus (DM) and osteoporosis[12]. A recent prospective cohort study demonstrated the reduction in ucOC level among the users of bisphosphonates. This reduction in ucOC level was not associated with changes in insulin and glucose levels or insulin to glucose ratio [13]. Another cohort study showed a reduction in risk to develop type 2 DM among the users of alendronate[14], but the result could be confounded by the difference of osteoporosis status between case and control group.

Since the launch of the Taiwanese National Health Insurance (NHI) in 1995, it had covered more than 98% of the population in 2005. We aimed to analyze the NHI database to find the association between the use of alendronate, a representative of bisphosphonates, and the incidence of newly diagnosed type 2 diabetes mellitus among osteoporotic patients.

## Patients and Methods

### Study design

The National Health Research Institute (NHRI) in Taiwan established the NHI Research Database (NHIRD) with the authorization from the NHI Administration, Ministry of Health and Welfare (MHW). The NHIRD selected a random sample with a representative population of 1,000,000 persons based on the year 2000 reimbursement data for public access. This database included information on ambulatory care, inpatient care, dental services, prescription drugs, medical institutions, and physician information. The encrypted personal identifications secured the confidentiality of individuals, precluding the possibility of the ethical violation of the data.

We used a retrospective cohort study design. The criterions were as follows:

#### Inclusion criteria

Subjects with a diagnosis of osteoporosis without DM from 2002 to 2006 were included. We then followed the subjects until they acquired a diagnosis of DM or to the end of 2009. The follow-up periods ranged from 4 (2006 cohort) to 8 (2002 cohort) years. All disease records in NHIRD contained one primary diagnosis and up to 4 secondary diagnoses by International Classification of Diseases, Ninth Revision, Clinical Modification (ICD-9CM) code. The ICD-9CM codes we used for the identification of subjects with osteoporosis were 733.0 (osteoporosis), 733.13 (pathologic fracture of vertebrae), 805, 806 (vertebral fracture), 820 (femoral neck fracture), 812.0, 812.2 (humerus fracture), 813.4 and 813.5 (wrist fracture). The code for DM was 250, which was assumed to be type 2 DM since it is acquired after age 45. We also collected the history of dyslipidemia (ICD-9CM code 272), hypertension (HTN, ICD-9CM code 401 to 405) as well as all diagnoses needed for Charlson Comorbid Index (CCI) as covariates.

#### Exclusion criteria

Subjects under age 45 or with multiple fractures were excluded. Multiple fractures were defined if they have one or more fracture codes other than those from our inclusion criteria. We also excluded subjects who ever used metformin or any statins at baseline.

We further divided these osteoporotic patients base on the treatments they received. By 2006, the MHW approved the following medications with indication for treatment (not prevention) of osteoporosis including calcitonin (1990), alendronate (1997), raloxifene (1999), and teriparatide (2003). Subjects not treated with any medications for osteoporosis were classified into the non-exposed group, and subjects who only used alendronate were classified into the exposed group. Subjects who were ever treated with other osteoporotic medications were excluded. Estrogen was not recommended as routine treatment for osteoporosis nowadays, but some patients might receive estrogen during 2002~2006 as treatment for osteoporosis. We excluded all patients who ever used estrogen from both groups. Other bisphosphonates, such as palmidronate or zolendronic acid, was approved for hypercalcemia but not osteoporosis. Subjects who were prescribed with bisphosphonates other than alendronate were not included as exposure group. Calcium and vitamin D supplements were allowed as concomitant medications.

### Statistical analysis

The subjects in exposed group were matched by the age and gender with those in non-exposed group in a 1:3 ratio. Pearson’s chi-square test was applied to compare discrete variables such as age, gender, geographic region, and comorbidity (DM, HTN and CCI) between exposed and non-exposed groups. The incidence rate of type 2 DM in both groups was calculated from count of DM patients divided by total person years. The Poisson regression with total person-time as an offset variable was applied to analyze the statistical difference between groups. In addition, the Kaplan-Meier curves was plotted to compare the incidence of type 2 DM in both groups during the study period, and the log-rank test was used to examine the significance of difference between groups. The P-value <0.05 was defined the statistical significance. The Statistical Analysis System (SAS) (version 9.2; SAS Institute, Inc, Cary, NC), was used to perform all statistical analyses.

### Ethics Statement

The institutional review board at the Chi Mei Medical Center approved this study.

## Results

**Fig 1:**
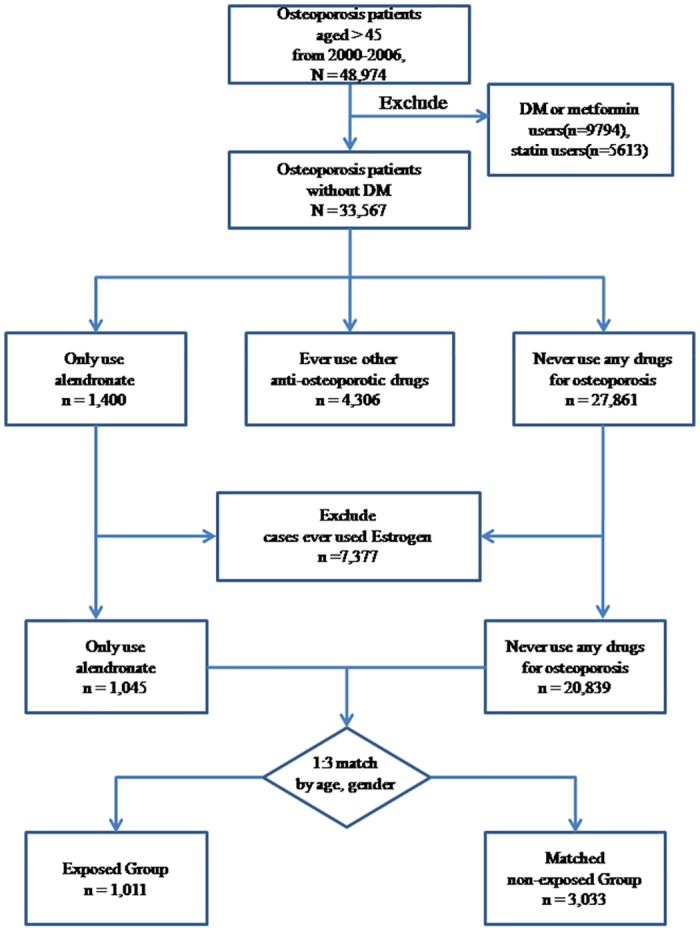
Flowchart of our study.

In our database, 48,974 osteoporotic patients more than 45 years were identified from 2002 to 2006. After excluding DM patients or subjects ever used metformin or statins, 33,567 subjects remained (Fig 1). Among them, 1,400 were prescribed alendronate but not other anti-osteoporotic drugs. Among the remainders, 27,861 patients were never prescribed any drug to treat osteoporosis. After excluding 7,377 subjects who were prescribed estrogen, 21,884 patients remained in the study sample, including 1,045 exposed subjects and 20,839 non-exposed subjects. After matching for age and gender, the exposed group contained 1,011 and the matched non-exposed group had 3,033 subjects (Fig 1). Our subjects distributed equally in different areas of Taiwan, and roughly 70% of them were more than 65 years and roughly three-fourth were female (Table 1). There was no significant between-group difference in the prevalence of comorbid conditions. The proportion of subjects who ever used glucocorticoids was also similar between groups. The proportion of vertebral fracture and femoral neck fracture were slightly higher in exposed group (Table 1).

**Table 1:** Demographic Characteristics of exposed and non-exposed Group.

	Non-exposed (N = 3033)	Exposed (N = 1011)	P-value
**Age**	70.39 (10.10)	70.40 (10.11)	0.9925
**Age (categorical)**			
<65	865 (28.52)	288 (28.49)	0.9840
≧65	2168 (71.48)	723 (71.51)	
**Gender**			
Female	2349 (77.45)	783 (77.45)	1.0000
Male	684 (22.55)	228 (22.55)	
**Geographic region**			
North or East	1243 (40.98)	395 (39.07)	0.5625
Center	605 (19.95)	208 (20.57)	
South	1185 (39.07)	408 (40.36)	
**Dyslipidemia**			
Yes	161 (5.31)	59 (5.84)	0.5219
No	2872 (94.69)	952 (94.16)	
**Hypertension (HTN)**			
Yes	1184 (39.04)	406 (40.16)	0.5274
No	1849 (60.96)	605 (59.84)	
**Dyslipidemia or HTN**			
Yes	1249 (41.18)	422 (41.74)	0.7539
No	1784 (58.82)	589 (58.26)	
**CCI score**			
0	1958 (52.69)	503 (49.75)	0.1935
1	747 (24.63)	275 (27.20)	
> = 2	688 (22.68)	233 (23.05)	
**Glucocorticoids use**			
Yes	303 (9.99)	123 (12.17)	0.0510
No	2730 (90.01)	888 (87.83)	
**ICD codes**			
Osteoporosis	2008 (66.21)	738 (73.00)	<0.0001
Pathologic fracture of vertebrae	17 (1.66)	15 (5.49)	0.0003
Vertebral fracture	361 (35.22)	120 (43.96)	0.0079
Femoral neck fracture	327 (31.90)	110 (40.29)	0.0091
Humerus fracture	153 (14.93)	31 (11.36)	0.1327
Wrist fracture	234 (22.83)	50 (18.32)	0.1089

Numbers (%) are reported for categorical variables.

Mean (SD) are reported for continuous variables.

Patients are with a median follow up of 5.84 years, and the inter-quartile range (IQR) is from 3.92 to 7.87.

CCI: Charlson co-morbidity index.

The incidence of DM among the non-exposed group was 397.08/10000 person year and the incidence among the exposed group is 329.15/10000 person year. The relative risk (RR) for developing DM in non-exposed group is 1.21 (95% confidence interval (CI): 1.03~1.41) (Table 2). The outcomes differed after we stratified the subjects by age, gender, comorbidities, and the frequency of prescriptions. The difference in DM risk was only significant among those younger than 65 years (RR: 1.59, 95%CI: 1.16~2.19), female subjects (RR: 1.19, 95%CI: 1.00~1.42), and those without dyslipidemia (RR: 1.20, 95%CI: 1.02~1.42), without hypertension (RR: 1.43, 95%CI: 1.13~1.80) or without both conditions (RR: 1.41, 95%CI: 1.11~1.80). Among exposed group, only who were prescribed alendronate for ≥3 times had significantly different DM incidence from non-exposed group (RR: 1.31, 95%CI: 1.07~1.60) (Table 2).

**Table 2:** Comparison for Incidence of type 2 DM between exposed and non-exposed group.

	Non-exposed group		Exposed group		IRR* (95%CI)	P- value
	Subjects	IR**	Subjects	IR**		
**Overall**	3033	397.08	1011	329.15	1.21(1.03–1.41)	**0.0204**
**Age**						
<65	865	411.94	288	258.98	1.59(1.16–2.19)	**0.0044**
≧65	2168	391.04	723	358.98	1.09(0.91–1.31)	0.3601
**Gender**						
Male	684	371.63	228	294.42	1.26(0.88–1.82)	0.2095
Female	2349	403.81	783	338.44	1.19(1.00–1.42)	0.0496
**Dyslipidemia**						
Yes	161	777.13	59	559.26	1.39(0.83–2.33)	0.2114
No	2872	379.41	952	315.23	1.20(1.02–1.42)	**0.0294**
**HTN**						
Yes	1184	492.72	406	472.31	1.04(0.84–1.30)	0.7027
No	1849	340.00	605	238.56	1.43(1.13–1.80)	**0.0029**
**Dyslipidemia or HTN**						
Yes	1249	516.09	422	479.50	1.08(0.87–1.33)	0.4940
No	1784	320.91	589	227.76	1.41(1.11–1.80)	**0.0056**
**CCI score**						
0	1598	340.33	503	281.01	1.21(0.96–1.53)	0.1127
1	747	423.55	275	309.59	1.37(1.00–1.87)	0.0501
> = 2	688	515.12	233	474.41	1.09(0.81–1.46)	0.5813
**Prescriptions**						
1~2	3033	397.08	399	370.34	1.07(0.85–1.34)	0.5463
> = 3	3033	397.08	612	303.81	1.31(1.07–1.60)	**0.0086**

*IRR: Incidence related risk

**IR: Incidence Rate, per 10,000 persons per year

**Fig 2:**
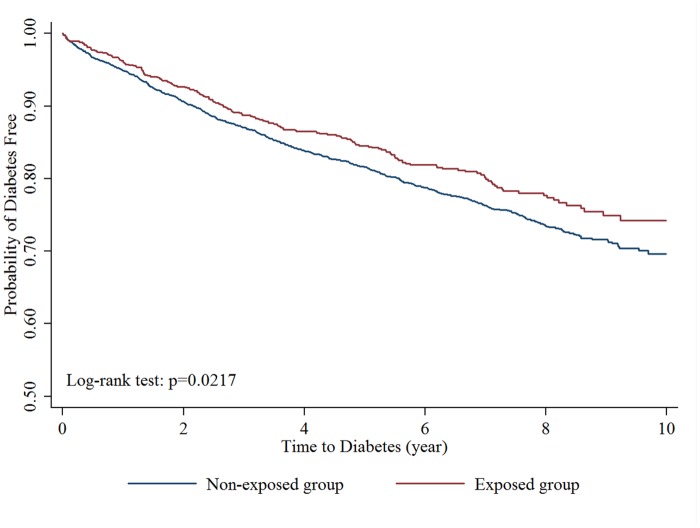
Kaplan-Meier curve of survival to newly diagnosed diabetes mellitus (exposed group vs non-exposed group).

**Fig 3:**
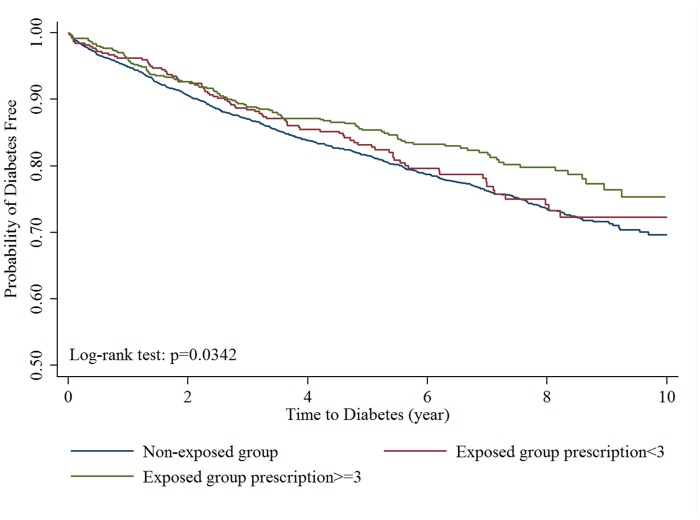
Kaplan-Meier curve of survival to newly diagnosed diabetes mellitus (exposed group classified by prescription times vs non-exposed group).

The Kaplan-Meier curves were shown in Figs 2 and 3. The log-rank test demonstrated significant lower cumulative risks for DM (P: 0.0217, Fig 2) during the follow-up period in exposed group. The median time to develop DM was 2.51 (Inter-quartile range (IQR): 1.17~4.65) years in non-exposed group and 2.60 (IQR: 1.30~4.81) years in exposed group, which was similar (p value: 0.5782) between two groups. The risk reduction in exposed group was only found in subjects who were prescribed alendronate for more than or equal to 3 times (Fig 3).

Table 3 showed the hazard ratio of each variable for incident DM in our study. After adjusted with age, gender, dyslipidemia and hypertension, the hazard ratio of alendronate non-exposure for DM was 1.23 (95% CI: 1.05~1.44) when compared with exposed subjects. The hazard ratio of hypertension and dyslipidemia for DM were 1.51 and 1.72. Table 4 showed that the higher risk for incident DM among alendronate non-exposed group was still persisted even after excluding new DM cases within 3 or 6 months after enrollments.

**Table 3:** Hazard Ratio for new onset type 2 DM (both group, N = 4044).

	Crude Hazard Ratio (95% C.I.)	P-value	Adjusted Hazard Ratio* (95% C.I.)	P-value
**Alendronate exposure**				
Exposed	1.00		1.00	
Non-exposed	1.20(1.03–1.41)	0.0219	1.23(1.05–1.44)	0.0123
**Age**				
<65	1.00		1.00	
≧65	1.03(0.89–1.19)	0.7311	0.94(0.81–1.09)	0.4008
**Gender**				
Male	1.00		1.00	
Female	1.12(0.95–1.32)	0.1832	1.11(0.94–1.31)	0.2155
**Dyslipidemia**				
No	1.00		1.00	
Yes	1.93(1.53–2.43)	<0.001	1.72(1.36–2.18)	<0.001
**HTN**				
No	1.00		1.00	
Yes	1.54(1.35–1.76)	<0.001	1.51(1.31–1.73)	<0.001

*Adjust for Age, Gender, dyslipidemia and HTN

**Table 4:** Sensitivity analysis of Hazard ratio for incident DM in both groups after excluding new DM cases within 3 or 6 months after enrollments.

	Alendronate exposure	Incident DM, n (%)	Adjusted* hazard ratio (95% CI)	P value
**Excluding DM < = 3 months**	Exposed group (n = 1000)	185(18.50)	1.00	0.0381
	Non-exposed group (n = 2974)	631(21.22)	1.19(1.01–1.40)	
**Excluding DM < = 6 month**	Exposed group (n = 988)	173(17.51)	1.00	0.0470
	Non-exposed group (n = 2932)	589(20.09)	1.19(1.00–1.41)	

*Adjust for Age, Gender, dyslipidemia and HTN

## Discussion

Our study showed a reduced risk of incident type 2 DM in the users of alendronate among osteoporotic patients. When compared with subjects who were prescribed alendronate, subjects who were not prescribed any anti-osteoporotic drug had a 21% increased risk to develop type 2 DM. The median time to develop DM was 2.51 years in non-exposed group and was similar to exposed group, 2.60 years. After stratification, the protective effect of alendronate was only significant among subjects younger than 65 years, and subjects without dyslipidemia or hypertension. Our results also implied a dose dependent effect showing that subjects who prescribed for ≥3 times of alendronate acquiring most protective effects.

Age, dyslipidemia and hypertension are all risk factors for DM. We performed subgroup analysis to detect possible interaction effects. The protective effect of alendronate was only significant in relatively young and healthy group indicate that the protective effect is not strong enough to nullify the risk for DM associated with those conditions. In fact, the hazards for DM caused by hypertension and dyslipidemia were both stronger than the protective effect of bisphosphonates in our study (Table 3). In addition, as statins used to treat dyslipidemia was found to be associated with an increased DM risk[15], we excluded statin users from our study to eliminate the confounding effect. A protective effect of alendronate was shown in most of our study subjects who had no dyslipidemia, and may be masked in the remainder because of the low case number. Age over 65 was not shown to be a significant risk for type 2 DM in Table 3, but the protective effect of alendronate could still be insignificant due to the higher prevalence of hypertension and dyslipidemia among subjects over 65 years.

DM was found to be associated with increased risk of osteoporosis[16]. However, it is not clear whether having osteoporosis is associated with increased risk of developing DM. In our cohort, the age-matched incidence of type 2 DM in the general population was 239. 40/10000 person year (data not shown), which was close to the results of a previous study [17] in Taiwan. The incidences of DM in both non-exposed and exposed group were significantly higher than that reported from general population, even the protective effect of alendronate was already taken into consideration. The underlying mechanism between osteoporosis and incident DM needs further exploration.

Vestergaard’s cohort study [14] in Denmark showed a reduction in risk to develop type 2 DM among users of alendronate, but their results were different from ours in some ways. First, their study classified subjects simply base on medication use status; these subjects who were not exposed to alendronate may or may not have osteoporosis. The interaction between osteoporosis and DM was not considered in this Danish study. Our study has the advantage for avoiding the confounding effect of osteoporosis on development of DM since subjects in both groups had osteoporosis as enrollment criteria. Second, our outcomes expanded the previous finding by demonstrating that alendronate may yield a dose-dependent protective effect for DM.

The major strength of our study is its population-based cohort design with relatively long follow-up periods so the generality and casual relationship can be ascertained. Moreover, we take into consideration about the frequency of prescriptions. The observed dose dependent protective effect also strengthens the causality determination from our study. We had detail information about the medications of study subjects so that we can successfully exclude statin and metformin users, which further strengthen our results. As the distribution of steroid users was similar in both groups, the confounding effects should be minimal due to the fact that we did not exclude glucocorticoid users. Subjects developed DM soon after inclusion was less likely associated with alendronate effect. Our findings were further strengthened since sensitivity analysis support the same result after excluding subjects developed DM within 6 months.

Selective estrogen receptor modulators (SERMs) were another class of anti-osteoporotic drug, which also reduce bone turnover and affect serum OC level in human[18]. Calcitonin reduces bone turnover, but its effect on serum OC is not clear. We applied the same model as alendronate to examine if there was the same effect from raloxifene (a SERM) and calcitonin on incidence of DM. Our results showed that exposure to both drugs were not associated with reduced number of incident DM (S1 and S2 Tables). The mechanism that makes alendronate different from other classes of anti-osteoporotic drug on glucose metabolism needs further exploration.

Our study is not without limitations. The overall prevalence of osteoporosis in our dataset was between 6.55% and 8.95% among female subjects, and was lower than that from other reports [19, 20]. Previous study suggested that prevalence of osteoporosis may be underestimated in studies use NHIRD [20]. However, the underestimation should be equally distributed between both groups and it would not affect the study outcome.

Another limitation came from the inadequate covariate information from the administrative data, such as body mass index (BMI), family history of diabetes and levels of physical activity. We were unable to adjust these potential covariates that were risk factors for on-set of diabetes. One major concern involved subject’s BMI. Subjects with higher BMI have higher incidence of DM but lower incidence of osteoporosis. This may raise the doubt that the subjects in exposed group may have lower BMI, which make them prone to be prescribed alendronate due to osteoporosis and meanwhile have lower incidence of DM. However, our exposed and non-exposed groups were both osteoporotic patients. The only difference between groups was the use of alendronate. BMI was not considered by the physician prescribing alendronate while BMI was not shown to affect or be affected by the use of alendronate. Therefore, the distributions of BMIs should be similar in both groups and we believed the impact for not collecting BMI data in our study is minimal.

Osteoporotic patients who were treated adequately may have more physical activity due to less clinical symptoms, such as pain and fracture, and consequently lead to lower risk of DM. This possible explanation for the protective effect of alendronate was similar to that from Maugeri’s study [12], but we were unable to exam it for lack of the information on physical activity.

Finally, we were not able to obtain serum OC levels with current set of administrative data. Serum OC was reported to influence glucose metabolism in both human and animal trials, and bisphosphonates were shown to influence OC. The findings in our study favor the hypothesis that bisphosphonates influence glucose metabolism in human, but the exact mechanism needs further exploration.

## Conclusions

Our study showed that alendronate might yield a protective effect on incidence of type 2 DM. This effect became less apparent in patients with older age, dyslipidemia or hypertension. The underlying mechanism needs further exploration and more prospective data are needed to confirm the observed findings.

## Supporting Information

S1 Table Demographic Characteristics of raloxifene users and matched non-exposed Group. (PDF)

S2 Table Demographic Characteristics of Calcitonin users and matched non-exposed Group. (PDF)
