# Supplementary material for: The Use of Alendronate Is Associated with a Decreased Incidence of Type 2 Diabetes Mellitus—A Population-Based Cohort Study in Taiwan
Source: PLoS One. 2015 Apr 13;10(4):e0123279. doi: 10.1371/journal.pone.0123279 (PMC4395231; doi:10.1371/journal.pone.0123279)
Supplement: S1 Table — (PDF) [file pone.0123279.s001.pdf]

S1 Table. Demographic Characteristics of raloxifene users and matched non-exposed Group

		Non-exposed group (N=1011)	Exposed group (N=337)	P-value
Age		71.00±10.35	71.01±10.36	0.9954
Age (categorical)				
	<65	258(25.52)	87(25.82)	0.9139
	≧65	753(74.48)	250(74.18)	
Gender				
	Female	984(97.33)	328(97.33)	1.0000
	Male	27(2.67)	9(2.67)	
Geographic region				
	North or East	411(40.65)	78(23.15)	<.0001
	Center	201(19.88)	92(27.30)	
	South	399(39.47)	167(49.55)	
Dyslipidemia				
	Yes	52(5.14)	14(4.15)	0.4662
	No	959(94.86)	323(95.85)	
Hypertension (HTN)				
	Yes	400(39.56)	139(41.25)	0.5853
	No	611(60.44)	198(58.75)	
Dyslipidemia or HTN				
	Yes	421(41.64)	143(42.43)	0.7987
	No	590(58.36)	194(57.57)	
CCI score				
	0	558(55.19)	168(49.85)	
	1	250(24.73)	101(29.97)	0.1348
	>=2	203(20.08)	68(20.18)	
Incident DM				
	Yes	226(22.35)	71(21.07)	0.6219
	No	785(77.65)	266(78.93)	

Numbers (%) are reported for categorical variables.
Mean (SD) or median(IQR) are reported for continuous variables.
CCI: Charlson co-morbidity index
