# Supplementary material for: The Use of Alendronate Is Associated with a Decreased Incidence of Type 2 Diabetes Mellitus—A Population-Based Cohort Study in Taiwan
Source: PLoS One. 2015 Apr 13;10(4):e0123279. doi: 10.1371/journal.pone.0123279 (PMC4395231; doi:10.1371/journal.pone.0123279)
Supplement: S2 Table — (PDF) [file pone.0123279.s002.pdf]

S2 Table. Demographic Characteristics of Calcitonin users and matched non-exposed Group

	Non-exposed group (N=3927)	Exposed group (N=1309)	P-value
Age	73.40±9.29	73.40±9.30	0.9977
Age (categorical)			
<65	663(16.88)	221(16.88)	1.0000
≧65	3264(83.12)	1088(83.12)	
Gender			
Female	2634(67.07)	878(67.07)	1.0000
Male	1293(32.93)	431(32.93)	
Geographic region			
North or East	1655(42.14)	614(46.91)	0.0010
Center	761(19.38)	265(20.24)	
South	1511(38.48)	430(32.85)	
Dyslipidemia			
Yes	194(4.94)	52(3.97)	0.1519
No	3733(95.06)	1257(96.03)	
Hypertension (HTN)			
Yes	1681(42.81)	597(45.61)	0.0767
No	2246(57.19)	712(54.39)	
Dyslipidemia or HTN			
Yes	1753(44.64)	619(47.29)	0.0955
No	2174(55.36)	690(52.71)	
CCI score			
0	1839(46.83)	563(43.01)	
1	1049(26.71)	356(27.20)	0.0272
>=2	1039(26.46)	390(29.79)	
Incident DM			
Yes	897(22.84)	307(23.45)	0.6491
No	3030(77.16)	1002(76.55)	

Numbers (%) are reported for categorical variables.
Mean (SD) or median(IQR) are reported for continuous variables.
CCI: Charlson co-morbidity index
